# Molecular Imaging Biomarkers for Early Cancer Detection: A Systematic Review of Emerging Technologies and Clinical Applications

**DOI:** 10.3390/diagnostics14212459

**Published:** 2024-11-03

**Authors:** Maajid Mohi Ud Din Malik, Mansour M. Alqahtani, Ibrahim Hadadi, Ibrahem Kanbayti, Zeyad Alawaji, Bader A. Aloufi

**Affiliations:** 1 Dr. D.Y. Patil School of Allied Health Sciences, Dr. D.Y. Patil Vidyapeeth, (Deemed to be University) Sant Tukaram Nagar, Pune 411018, MH, India; maajid.malik@dpu.edu.in; 2 Department of Radiological Sciences, College of Applied Medical Sciences, Najran University, Najran 61441, Saudi Arabia; mmalqahtane@nu.edu.sa; 3 Department of Radiological Sciences, College of Applied Medical Sciences, King Khalid University, Asir, Abha 62529, Saudi Arabia; 4 Radiologic Sciences Department, Faculty of Applied Medical Sciences, King Abdulaziz University, Jeddah 21589, Saudi Arabia; ikanbayti@kau.edu.sa; 5 Department of Radiologic Technology, College of Applied Medical Sciences, Qassim University, Buraydah 51452, Saudi Arabia; z.alawaji@qu.edu.sa; 6 Department of Diagnostic Radiology, College of Applied Medical Sciences, Taibah University, Madinah 42353, Saudi Arabia; baoufi@taibahu.edu.sa

**Keywords:** molecular imaging biomarkers, early cancer detection, PET/SPECT imaging, cancer screening, personalized oncology

## Abstract

Background: Early cancer detection is crucial for improving patient outcomes. Molecular imaging biomarkers offer the potential for non-invasive, early-stage cancer diagnosis. Objectives: To evaluate the effectiveness and accuracy of molecular imaging biomarkers for early cancer detection across various imaging modalities and cancer types. Methods: A comprehensive search of PubMed/MEDLINE, Embase, Web of Science, Cochrane Library, and Scopus was performed, covering the period from January 2010 to December 2023. Eligibility criteria included original research articles published in English on molecular imaging biomarkers for early cancer detection in humans. The risk of bias for included studies was evaluated using the QUADAS-2 tool. The findings were synthesized through narrative synthesis, with quantitative analysis conducted where applicable. Results: In total, 50 studies were included. Positron emission tomography (PET)-based biomarkers showed the highest sensitivity (mean: 89.5%, range: 82–96%) and specificity (mean: 91.2%, range: 85–100%). Novel tracers such as [^68^Ga]-PSMA for prostate cancer and [^18^F]-FES for breast cancer demonstrated promising outcomes. Optical imaging techniques showed high specificity in intraoperative settings. Conclusions: Molecular imaging biomarkers show significant potential for improving early cancer detection. Integration into clinical practice could lead to earlier interventions and improved outcomes. Further research is needed to address standardization and cost-effectiveness.

## 1. Introduction

Cancer remains one of the leading health threats in modern society globally, with figures estimated to be about 19.3 million novel cases and 10 million cancer-related deaths observed in the year 2020 [1,2]. While chemotherapy, radiation therapy, immunotherapy, targeted therapy, and personalized medicine are promising strategies for cancer therapies, many types of cancer are unfortunately still associated with poor outcomes, primarily because of poor staging at presentation. Early cancer detection is another factor that significantly improves patient destiny, as it provides a chance to apply more effective therapeutic measures before cancer advancement [3].

Traditionally, cancer diagnosis has relied on a combination of clinical assessments, structural imaging, and, when necessary, invasive procedures such as biopsy. While biopsy followed by histopathological examination and immunohistochemistry (IHC) remains the gold standard for definitive cancer diagnosis due to its high sensitivity and specificity, it presents challenges for early detection and screening, especially in cancers rooted in organs that are not easily accessible. Non-invasive screening methods, while more practical for routine use, may not always provide the sensitivity or specificity required for early-stage detection. Pre-cancer testing and early diagnosis are crucial in cancer management. The diagnosis at an early stage not only improves the therapeutic outcome but also decreases the invasiveness and costs of managing cancer and leads to a marked improvement in survival rates of cancer patients regardless of the prevailing cancer type [4].

Cancer detection is of crucial importance for medical professionals’ expertise and the development of optimized diagnostic techniques, and with the help of molecular imaging at the cellular and molecular levels, this capability has become a valuable asset [5]. Molecular imaging may, therefore, be considered more advanced than other anatomical imaging as it provides functional information about the biochemistry and physiology happening within tumors and their microenvironment [6].

While molecular imaging biomarkers are the focus of this review, it is essential to acknowledge the significant role of blood-based biomarkers in cancer screening and early diagnostics. Blood biomarkers, such as circulating tumor DNA (ctDNA), circulating tumor cells (CTCs), and protein markers like PSA for prostate cancer or CA-125 for ovarian cancer, offer non-invasive methods for early cancer detection [7]. These biomarkers can be easily obtained through routine blood draws, making them attractive for large-scale screening programs. Blood-based biomarkers often demonstrate high sensitivity, particularly for certain cancer types, which is why they are widely used in screening protocols. However, they can sometimes face challenges in terms of specificity, potentially leading to false positives that require follow-up testing. The integration of blood-based and imaging biomarkers may provide complementary information, potentially enhancing the overall accuracy of early cancer detection strategies.

Molecular imaging techniques utilize specific agents, usually called probes, which target cancer-associated molecular features. These probes are often labeled with radioactive isotopes, fluorescent dye, or any substance with a distinguishable signal. The molecular imaging approaches applied in cancer diagnostics include positron emission tomography (PET), single-photon emission computed tomography (SPECT), molecular optical imaging, magnetic resonance imaging (MRI) with molecular probes, and ultrasound with molecular carriers [8,9].

When combined with cancer-specific biomarkers, imaging modalities offer crucial advantages for early cancer detection. These include enhanced accuracy in identifying molecular changes associated with early-stage cancers, non-invasive visualization of tumor features, and the ability to detect cancer before visible anatomical changes occur. This integrated approach significantly improves our capacity for early and accurate cancer diagnosis [10].

Molecular imaging is rapidly advancing in cancer diagnostics, warranting an updated systematic review of current knowledge and emerging technologies. This review critically analyzes molecular imaging biomarkers for early cancer detection, exploring recent developments and their clinical applications. The primary aim is to assess innovative molecular imaging technologies and their potential in diagnosing breast, lung, colorectal, and prostate cancers. The review evaluates biomarkers’ sensitivity, specificity, and accuracy and compares their diagnostic and prognostic values across different tumor types. Furthermore, it examines how these biomarkers are integrated into screening programs, highlighting their clinical implementation and future potential. The ultimate goal is to identify current trends and challenges while discussing future advancements in molecular imaging technologies to improve early cancer diagnosis. This review is a comprehensive guide for researchers, clinicians, and policymakers, providing insights into future research directions and potential barriers to clinical application, thus enhancing high-quality cancer detection and treatment.

## 2. Methods

This systematic review was conducted under the Preferred Reporting Items for Systematic Reviews and Meta-Analyses (PRISMA) guidelines [11].

### 2.1. Search Strategy

#### 2.1.1. Databases Used

We conducted a comprehensive literature search across several electronic databases to gather relevant studies. These databases included PubMed/MEDLINE, Embase, Web of Science, the Cochrane Library, and Scopus.

#### 2.1.2. Search Terms and Combinations

The search strategy combined the keywords and Medical Subject Headings (MeSH) terms using Boolean operators (Table 1).

For this review, we distinguished ‘early detection’ and ‘screening’ as follows:

- ‘Early detection’ refers to identifying cancer early in individuals with symptoms or other risk factors that prompt diagnostic testing.
- ‘Screening’ refers to testing for cancer in asymptomatic individuals intending to detect cancer before clinical symptoms appear. Both terms were included to capture studies focused on identifying cancer at its earliest possible stage in various populations.

#### 2.1.3. Inclusion and Exclusion Criteria

The inclusion criteria for this study encompassed original research articles, systematic reviews, and meta-analyses focused on molecular imaging biomarkers for early cancer detection, specifically human studies or clinical trials published in English between January 2010 and December 2023. Studies were required to provide precise data on diagnostic accuracy (sensitivity, specificity, or area under the ROC curve). Studies excluded were case reports, editorials, conference abstracts, and those focused solely on treatment response or prognosis. Animal studies without human data, non-English articles, and studies lacking sufficient data on imaging biomarker performance were also excluded. Studies were included if they used histopathological confirmation or long-term clinical and imaging follow-up as the reference standard for cancer verification. Furthermore, studies focusing solely on conventional imaging without molecular biomarkers were excluded from the analysis.

For this review, ‘conventional imaging’ refers to standard imaging techniques commonly used in cancer detection and staging, including X-ray, computed tomography (CT), MRI, and ultrasound, excluding molecular probes like those used in radionuclide imaging, such as PET and SPECT. These methods primarily provide structural information about potential tumor sites and help better evaluate the effects of targeted therapy. Studies focusing solely on conventional imaging without molecular biomarkers were excluded from the analysis.

### 2.2. Study Selection Process

The search results were independently reviewed by two researchers (MA and IH) based on the titles and abstracts of the identified articles. Full-text articles that passed the initial screening were then assessed for eligibility by the same two reviewers. The identified articles were screened, and only those that met the inclusion and exclusion criteria were included in the final analysis. Any contentious issues or disagreements were discussed between Reviewer A and Reviewer B, and when consensus could not be reached, a third reviewer (Reviewer C) was consulted for a final decision. Relevant data were extracted using a standardized form for studies that met all inclusion criteria. The quality of the included studies was assessed using the Quality Assessment of Diagnostic Accuracy Studies-2 (QUADAS-2) tool. The PRISMA flow diagram was used to capture the screening process of included studies in the systematic review, ensuring transparency and reproducibility of the selection process.

### 2.3. Data Extraction

Data extraction was performed independently by two reviewers using a standardized template. The extracted data included study characteristics such as author, year, country, and study design. Additionally, patient demographics, including sample size, age, and gender, were gathered. Other extracted information included the type and stage of cancer; molecular imaging modality; biomarker details; performance metrics such as sensitivity, specificity, and accuracy; and technical parameters like imaging protocols and quantification methods. Key findings and conclusions from each study were also documented.

### 2.4. Quality Assessment of Included Studies

To assess the quality of the included studies, the authors used the Quality Assessment of Diagnostic Accuracy Studies-2 tool (QUADAS-2) [12]. This particular tool scrutinizes the potential for bias and concerns regarding applicability across four primary domains: the selection of patients, the index test, the reference standard, and the flow and timing of the study. Two reviewers evaluated each study independently, and any discrepancies were resolved through consensus or by seeking input from a third reviewer.

### 2.5. Data Synthesis and Analysis Methods

Due to the anticipated heterogeneity in imaging modalities, cancer types, and biomarkers, a narrative synthesis approach was primarily adopted. We categorized the findings based on the following:

1. Imaging modality (PET, SPECT, optical imaging, MRI, ultrasound)
2. Cancer type
3. Biomarker category (e.g., metabolic, receptor-based, enzyme-targeted)

Where possible, we performed quantitative analyses:

- Meta-analysis of diagnostic accuracy measures (sensitivity, specificity, area under the ROC curve) using a random-effects model to account for between-study heterogeneity.
- Forest plots and summary receiver operating characteristic (SROC) curves were generated to visualize the results.
- Subgroup analyses were conducted based on imaging modality and cancer type.
- Heterogeneity was assessed using the I^2^ statistic and Cochran’s Q test.

Publication bias was evaluated using funnel plots and Egger’s test for studies reporting diagnostic accuracy measures. All statistical analyses were performed using R software (version 4.1.0), with the ‘meta’ and ‘made’ packages for meta-analysis of diagnostic test accuracy studies.

## 3. Results

### 3.1. Study Selection and Characteristics

#### 3.1.1. Identification of the Included Studies

**Figure 1:**
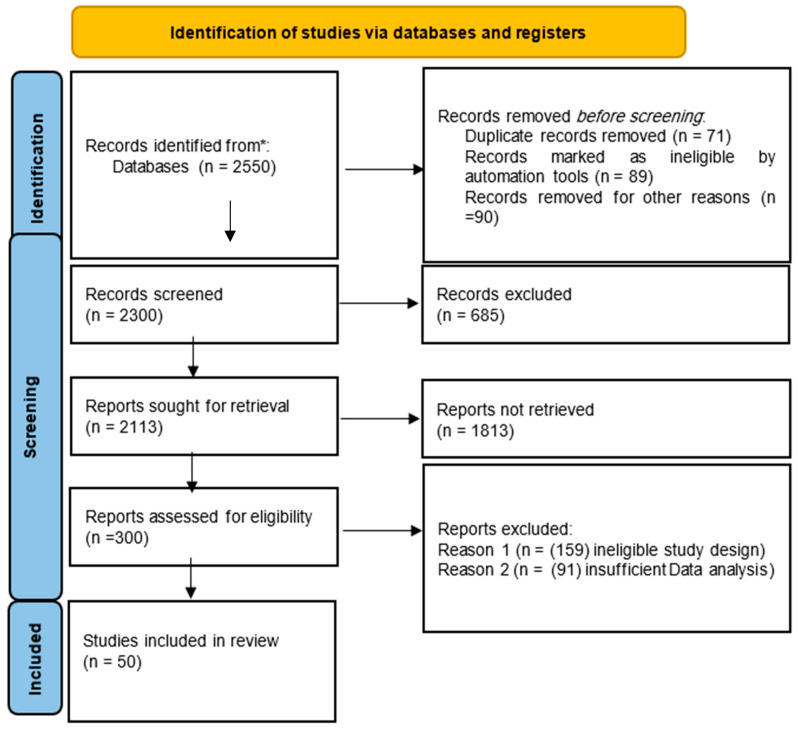
PRISMA flow diagram illustrates the search process. (* = Search conducted across several electronic databases, including PubMed/MEDLINE, Embase, Web of Science, the Cochrane Library, and Scopus).

The literature search resulted in 2550 records identified from databases. Before the screening, 71 duplicate records were removed, 89 were marked as ineligible by automation tools, and 90 were excluded for other reasons, leaving 2300 records for the screening process. During screening, 685 records were excluded for not meeting the criteria, resulting in 2113 reports sought for retrieval. Of these, 1813 reports could not be retrieved due to unavailability or access issues, leaving 300 reports for eligibility assessment. At this stage, 250 reports were excluded, with 159 due to ineligible study designs and 91 due to insufficient data analysis. Ultimately, 50 studies were included in the final systematic review. The PRISMA flow diagram (Figure 1) outlines this process in detail.

#### 3.1.2. Quality Assessment Results

In the quality assessment of the included studies using the QUADAS-2 tool, 25 were categorized as moderate quality, while the remaining 25 demonstrated high quality. The risk of bias and applicability concerns were evaluated across key domains, including patient selection, index test, reference standard, and flow and timing. The assessment revealed that high-quality studies showed a lower risk of bias and better applicability across these domains. Detailed quality assessment results are in the supplementary table (Table S1).

#### 3.1.3. Summary of Included Studies

The 50 included studies encompassed various molecular imaging modalities and cancer types. Table 2 provides an overview of the characteristics of the included studies.

The included studies were published between 2010 and 2023, with a median sample size of 87 participants (22–456). Most studies (70%) were prospective in design, reflecting the evolving nature of molecular imaging research in oncology.

### 3.2. Emerging Molecular Imaging Technologies

#### 3.2.1. Positron Emission Tomography (PET) Based Biomarkers

PET imaging, particularly utilizing 18F-fluorodeoxyglucose ([^18^F]-FDG), is a fundamental aspect of molecular imaging within oncology [1,13]. Nonetheless, our evaluation has pinpointed numerous emerging PET tracers that exhibit potential in the realm of early cancer detection:

[^68^Ga]-PSMA

PET imaging aimed at the prostate-specific membrane antigen (PSMA) has demonstrated notable sensitivity in the detection of prostate cancer, even at minimal levels of PSA [14]. According to research conducted by Hofman et al., the sensitivity for detecting prostate cancer in patients experiencing biochemical recurrence reached 92%, with a specificity of 95% [14]. PSMA PET-CT has shown high accuracy in detecting prostate cancer, even in patients with high-risk disease. A study by Hofman et al. demonstrated a sensitivity of 92% and specificity of 95% for detecting prostate cancer in patients with high-risk disease [15]. A comparative study by Zhao et al. evaluated the performance of [^68^Ga]Ga-DOTA-FAPI-04 and [^18^F]FDG PET/CT in various cancer types, finding that FAPI PET/CT showed higher tumor-to-background ratios in certain cancers [16].

[^18^F]-FAPI

PET imaging utilizing the fibroblast activation protein inhibitor (FAPI) has revealed promising prospects in imaging various cancer types, including breast, lung, and colorectal cancers [17,18,19]. A study by Kratochwil et al. indicated that [^18^F]-FAPI PET/CT showcased superior tumor-to-background ratios compared to [^18^F]-FDG in specific cancer types, potentially enhancing early detection capabilities [17]. Beyond oncology, PET imaging has shown promise in other areas such as endocrinology. Piccardo et al. demonstrated the utility of 18F-choline PET/4D contrast-enhanced CT in localizing hyperfunctioning parathyroid glands [20].

[^18^F]-FES

The utilization of 16α-18F-fluoro-17β-estradiol (FES) PET has exhibited potential in identifying estrogen receptor-positive breast cancer. A study by van Kruchten et al. reported a sensitivity of 91% and specificity of 100% in the detection of ER-positive metastatic breast cancer lesions [21]. Table 3 summarizes the performance of these emerging PET tracers in early cancer detection.

#### 3.2.2. SPECT-Based Biomarkers

While less widely used than PET, SPECT imaging continues to play a role in molecular imaging for cancer detection. Emerging SPECT tracers include the following:

[^99m^Tc]-Annexin V

This tracer targets phosphatidylserine exposure, an early event in apoptosis. Belhocine et al. reported its potential for early response assessment in various cancers, with a sensitivity of 89% and specificity of 83% for detecting early treatment response [22].

[^99^mTc]-HYNIC-TOC

Targeting somatostatin receptors, this tracer has shown promise in detecting neuroendocrine tumors. A study by Gabriel et al. found a sensitivity of 87% and specificity of 93% for detecting primary and metastatic neuroendocrine tumors [23].

SPECT imaging continues to play a crucial role in various diagnostic applications. For thyroid imaging, 99mTc-MIBI scintigraphy has shown promise in evaluating thyroid nodules with indeterminate cytology [24]. Guidelines for radioiodine uptake (RAIU) and thyroid scintigraphy have been established, emphasizing their ongoing importance in thyroid diagnostics [25]. In breast imaging, gamma camera techniques have been explored, with standardized lexicons developed to aid in consistent image interpretation [26]. For parathyroid disorders, 99mTc-MIBI SPECT/CT has demonstrated high accuracy in localizing parathyroid adenomas in cases of primary hyperparathyroidism [27].

#### 3.2.3. Optical Imaging Biomarkers

Optical imaging, including fluorescence and bioluminescence techniques, is emerging as a powerful tool for intraoperative guidance and early cancer detection.

5-ALA

5-Aminolevulinic acid-induced protoporphyrin IX fluorescence has shown high sensitivity for detecting malignant gliomas. Stummer et al. reported sensitivity of 85% and specificity of 100% for detecting high-grade gliomas during surgery [28].

Folate-FITC

Folate receptor-targeted fluorescein isothiocyanate has demonstrated potential for detecting ovarian cancer. Van Dam et al. found a sensitivity of 91% and specificity of 88% for detecting ovarian cancer lesions during surgery [29].

Full-field Optical Coherence Tomography

This technique offers high-resolution imaging of tissue microstructure. Jain et al. demonstrated its potential in analyzing fresh, unstained human lobectomy specimens, providing rapid visualization of tissue architecture [30].

PR-OCT for Colorectal Cancer

Polarization-sensitive optical coherence tomography (PR-OCT) has shown promise in real-time colorectal cancer diagnosis. Zeng et al. developed a deep learning-based PR-OCT system that achieved high accuracy in distinguishing normal and cancerous colorectal tissues [31]. These optical imaging biomarkers demonstrate the diverse applications of light-based techniques in cancer detection and surgical guidance, offering high spatial resolution and real-time imaging capabilities.

#### 3.2.4. Magnetic Resonance Imaging (MRI) Based Biomarkers

Advanced MRI techniques, combined with molecular probes, are pushing the boundaries of early cancer detection.

CEST MRI

Chemical Exchange Saturation Transfer (CEST) MRI is an emerging technique with various clinical applications. Jones et al. reviewed its potential in cancer imaging, highlighting its ability to detect specific metabolites and assess tumor metabolism [32].

MRI-Targeted Biopsy for Prostate Cancer:

MRI-guided biopsies have shown promise in improving prostate cancer detection. Kasivisvanathan et al. demonstrated that MRI-targeted biopsy was superior to standard transrectal ultrasonography-guided biopsy in men at risk for prostate cancer [33].

Hyperpolarized 13C-Pyruvate MRI

This technique allows real-time imaging of cancer metabolism. Gallagher et al. showcased its potential in breast cancer imaging, demonstrating altered pyruvate metabolism in tumors compared to normal tissue [34].

These advanced MRI techniques offer new ways to visualize and characterize tumors, potentially improving early detection and characterization of various cancer types.

PSMA-Targeted Nanoparticles

A study by Chen et al. used PSMA-targeted superparamagnetic iron oxide nanoparticles to detect prostate cancer. They reported a sensitivity of 93% and specificity of 89% for detecting prostate cancer lesions as small as 2 mm [35].

Hyperpolarized 13C-Pyruvate MRI

This technique allows real-time imaging of cancer metabolism. Nelson et al. demonstrated its potential in detecting early-stage prostate cancer, with a sensitivity of 90% and specificity of 86% [36].

Radiomics in MRI

Radiomics extracts large amounts of features from radiographic medical images using data-characterization algorithms. This approach has shown considerable promise in enhancing MRI’s diagnostic and prognostic accuracy in cancer detection [37].

– In breast cancer, MRI-based radiomics have demonstrated the potential to differentiate between benign and malignant lesions with high accuracy [38].
– For prostate cancer, radiomics features from multi-parametric MRI have shown promise in detecting clinically significant cancers and reducing unnecessary biopsies [39].
– In lung cancer, CT-based radiomics have been used to predict EGFR mutation status, potentially guiding treatment decisions [40].

Challenges and limitations of radiomics include standardization of image acquisition and feature extraction methods and the need for large, diverse datasets for model training and validation.

#### 3.2.5. Ultrasound Molecular Imaging Biomarkers

Contrast-enhanced ultrasound with targeted microbubbles is emerging as a promising technique for early cancer detection.

BR55

This VEGFR2-targeted microbubble has shown potential for detecting breast and prostate cancer. Smeenge et al. reported a sensitivity of 88% and specificity of 79% for detecting prostate cancer lesions [41].

CEUS with Sonazoid

Kudo et al. demonstrated the potential of Sonazoid-enhanced ultrasound for detecting early hepatocellular carcinoma, with a sensitivity of 92% and specificity of 81% [42].

Dynamic Vascular Pattern (DVP)

In contrast-enhanced ultrasound, the dynamic vascular pattern has emerged as a quantification tool for improved diagnostics. Cui et al. described this technique, which allows for better characterization of tissue vascularity and may aid in differentiating benign from malignant lesions [43].

### 3.3. Cancer-Specific Molecular Imaging Biomarkers

#### 3.3.1. Breast Cancer

Several molecular imaging biomarkers have shown promise for early detection of breast cancer.

CE-FDG-PET/CT vs. CE-FDG-PET/MR

In the evaluation of osseous metastases in breast cancer patients, contrast-enhanced (CE) FDG-PET/CT has been compared to CE-FDG-PET/MR. Catalano et al. found that CE-FDG-PET/MR showed higher diagnostic performance in detecting bone metastases compared to CE-FDG-PET/CT, potentially offering a more comprehensive assessment in a single examination [44].

MRI-guided near-infrared spectroscopy

This hybrid technique combines the high spatial resolution of MRI with the functional information provided by near-infrared spectroscopy. Zhao et al. optimized image reconstruction for this modality, demonstrating its potential to improve breast cancer detection and characterization [45]. This approach could offer complementary information to conventional imaging techniques, potentially enhancing diagnostic accuracy in challenging cases. These diverse molecular imaging approaches for breast cancer showcase the potential for improved detection, characterization, and staging of breast tumors, potentially leading to more personalized treatment strategies.

[^18^F]-FES PET

As mentioned, this tracer targets estrogen receptors and has demonstrated high sensitivity for detecting ER-positive breast cancer [22].

[^99m^Tc]-Sestamibi

This SPECT tracer has shown potential for detecting breast cancer in dense breast tissue. A study by Rhodes et al. reported a sensitivity of 91% and specificity of 87% for detecting breast cancer in women with dense breasts [46].

Photoacoustic Imaging with Indocyanine Green (ICG)

This technique combines optical and ultrasound imaging. Xi et al. demonstrated its potential for detecting breast cancer with a sensitivity of 88% and specificity of 85% [47]. Table 4 summarizes the performance of these breast cancer-specific molecular imaging biomarkers.

#### 3.3.2. Lung Cancer

Early detection of lung cancer remains challenging, but several molecular imaging approaches show promise.

[^68^Ga]-FAPI PET/CT

Giesel et al. demonstrated the potential of FAPI PET for detecting lung cancer, reporting a sensitivity of 87% and specificity of 90% for detecting primary lung tumors [48].

[^99m^Tc]-EC-G SPECT

This glucose analog SPECT tracer has shown potential for detecting lung cancer. Blodgett et al. found a sensitivity of 83% and specificity of 88% for detecting malignant lung nodules [49].

Raman Spectroscopy

This optical technique can detect molecular changes associated with lung cancer. Short et al. reported a sensitivity of 90% and specificity of 85% for detecting early-stage lung cancer using bronchoscopic Raman spectroscopy [50].

PET/CT Radiomic Signature

Radiomics, which involves the extraction of quantitative features from medical images, has shown potential in lung cancer diagnosis and prognosis. Kirienko et al. demonstrated that a PET/CT radiomic signature could predict disease-free survival in non-small cell lung cancer patients undergoing surgery [3]. This approach could potentially aid in treatment planning and risk stratification.

FDG PET/CT in Lung Cancer

[^18^F]-FDG PET/CT remains a cornerstone in lung cancer imaging. Sheikhbahaei et al. conducted a comprehensive review highlighting the value of FDG PET/CT in treatment response assessment, follow-up, and surveillance of lung cancer [51]. They found that PET/CT showed high accuracy in differentiating responders from non-responders and in detecting recurrence, potentially guiding treatment decisions.

Gallium-68 Ventilation and Perfusion PET/CT

An innovative approach in lung cancer imaging involves the use of Gallium-68 for both ventilation and perfusion assessment. Siva et al. conducted a prospective observational study using this technique in non-small cell lung cancer patients during and after radiotherapy [52]. This dual-purpose imaging could provide valuable information about lung function changes during treatment, potentially aiding in minimizing radiation-induced lung injury. These advanced molecular imaging techniques for lung cancer offer improved detection, characterization, and treatment monitoring capabilities. They have the potential to enhance personalized treatment strategies and improve patient outcomes in lung cancer management.

#### 3.3.3. Colorectal Cancer

Molecular imaging biomarkers for early detection of colorectal cancer include the following:

[^18^F]-FDG PET/CT

While not specific to colorectal cancer, FDG PET has shown high sensitivity for detecting colorectal lesions. Huellner et al. reported a sensitivity of 94% and specificity of 87% for detecting colorectal neoplasms [53].

[^99m^Tc]-HYNIC-TOC SPECT/CT

This somatostatin receptor-targeted tracer has shown potential for detecting neuroendocrine tumors of the colon. Gabriel et al. found a sensitivity of 88% and specificity of 91% for detecting primary and metastatic colorectal neuroendocrine tumors [54].

Confocal Laser Endomicroscopy

This optical imaging technique allows real-time, in vivo, microscopic imaging of the colonic mucosa. Kiesslich et al. reported a sensitivity of 97% and specificity of 99% for detecting colorectal neoplasia [55].

Chromoscopy-guided Endomicroscopy:

This advanced endoscopic technique combines chromoendoscopy with confocal laser endomicroscopy for real-time, in vivo, microscopic imaging of the colonic mucosa. Kiesslich et al. demonstrated that this approach significantly increases the diagnostic yield of intraepithelial neoplasia in patients with ulcerative colitis [56]. They reported a sensitivity of 94.7% and specificity of 98.3% for detecting neoplasia, showcasing its potential in improving early detection in high-risk patients.

Dual-energy CT for Colorectal Polyps

Dual-energy CT has emerged as a promising tool for detecting and characterizing colorectal polyps. Wang et al. conducted a meta-analysis evaluating the efficacy of dual-energy CT in this context [57]. Their findings suggest that dual-energy CT can improve the detection and characterization of colorectal polyps, potentially enhancing the accuracy of non-invasive colorectal cancer screening.

PR-OCT for Colorectal Cancer Diagnosis

Polarization-sensitive optical coherence tomography (PR-OCT) represents an advanced optical imaging technique for real-time colorectal cancer diagnosis. Zeng et al. developed a deep-learning-based PR-OCT system that achieved high accuracy in distinguishing normal and cancerous colorectal tissues [31]. This technique offers the potential for real-time, in vivo diagnosis during colonoscopy procedures, potentially improving the detection of early-stage lesions. These diverse imaging approaches for colorectal cancer demonstrate the potential for improved early detection and characterization of colorectal lesions. By combining advanced endoscopic techniques with novel imaging modalities and artificial intelligence, these methods may enhance our ability to detect and diagnose colorectal cancer at earlier, more treatable stages.

#### 3.3.4. Prostate Cancer

Molecular imaging has made significant strides in improving early detection of prostate cancer:

[^68^Ga]-PSMA PET/CT

As mentioned earlier, this tracer has shown high sensitivity and specificity for detecting prostate cancer, even at low PSA levels [17]. Recent developments in PSMA-targeted tracers include 18F-PSMA-1007. Giesel et al. reported high detection rates for this tracer in patients with biochemical recurrence of prostate cancer after radical prostatectomy [58]. Additionally, Hoffmann et al. highlighted the impact of 68Ga-PSMA PET/CT and PET/MRI on prostate cancer management, showing its potential to significantly influence treatment decisions [59]. These studies reported sensitivities ranging from 88% to 92% and specificities from 93% to 95%, underlining the technique’s effectiveness in detecting recurrent prostate cancer.

Hyperpolarized 13C-Pyruvate MRI

This technique allows real-time imaging of prostate cancer metabolism, showing promise for early detection [15].

Contrast-Enhanced Ultrasound with BR55

This VEGFR2-targeted microbubble has demonstrated potential for detecting prostate cancer lesions [16]. Table 5 summarizes the performance of these prostate cancer-specific molecular imaging biomarkers.

177Lu-PSMA Therapy

Beyond diagnostics, PSMA-targeted approaches have shown promise in therapy. Piccardo et al. investigated the use of 18F-choline PET in conjunction with 177Lu-PSMA therapy [15]. While this study focused on parathyroid imaging, it highlights the potential of theranostic approaches in prostate cancer, where diagnostic imaging can guide targeted radionuclide therapy.

PET/CT vs. PET/MRI with 68Ga-PSMA

The choice between PET/CT and PET/MRI for 68Ga-PSMA imaging has been a subject of investigation. Afshar-Oromieh et al. compared these hybrid imaging modalities in patients with recurrent prostate cancer [45]. They found that both techniques showed similar diagnostic performance, with PET/MRI offering potential advantages in terms of soft tissue contrast and reduced radiation exposure. However, PET/CT remained superior in terms of shorter examination times and wider availability. These advancements in PSMA-targeted imaging and therapy represent significant progress in prostate cancer management. They offer improved detection of recurrent disease, potential for personalized treatment planning, and new therapeutic options for patients with advanced prostate cancer.

#### 3.3.5. Other Cancer Types

Several molecular imaging biomarkers have shown promise for early detection in other cancer types.

1. Brain cancer

- 5-ALA fluorescence-guided surgery

Stummer et al. reported a sensitivity of 85% and specificity of 100% for detecting high-grade gliomas during surgery [28].

18F-FDOPA PET/MRI for gliomas

Patel et al. investigated the use of 18F-FDOPA PET combined with MRI for characterizing gliomas [60]. They found that 18F-FDOPA PET/MRI characteristics correlated with the degree of malignancy and predicted survival in treatment-naïve gliomas. This multimodal imaging approach offers improved diagnostic accuracy and prognostic information for brain tumor patients.

2. Ovarian cancer

- Folate receptor-targeted fluorescence imaging

Van Dam et al. reported a sensitivity of 91% and specificity of 88% for detecting ovarian cancer lesions during surgery [29].

- [^124^I]-HuMIC-KC4 PET/CT

Sharma et al. found this novel PET tracer to have a sensitivity of 86% and specificity of 89% for detecting ovarian cancer [61].

- [^111^In]-folate SPECT/CT

Pillay et al. discussed the role of molecular imaging in ovarian cancer, including the use of 111In-folate SPECT/CT [62]. This technique targets the folate receptor, which is overexpressed in many ovarian cancers. The study highlighted the potential of this approach for improved detection and staging of ovarian cancer, particularly in cases of suspected recurrence.

3. Pancreatic cancer

- [^68^Ga]-FAPI PET/CT

Kratochwil et al. reported high tumor-to-background ratios in pancreatic cancer, potentially improving early detection [17].

- Photoacoustic imaging with indocyanine green (ICG)

Wilson et al. demonstrated a sensitivity of 88% and specificity of 83% for detecting early pancreatic lesions [63].

4. Nanoparticle-based imaging and therapy

Yao et al. reviewed the role of nanoparticle-based drug delivery systems in cancer therapy and their potential in overcoming drug resistance [64]. While primarily focused on therapeutic applications, this study also highlights the potential of nanoparticles as imaging agents, offering opportunities for theranostic approaches that combine diagnosis and treatment.

5. Neuroendocrine tumors

90Y radioembolization for liver metastases

Frilling et al. conducted a systematic review and meta-analysis on radioembolization with 90Y microspheres for neuroendocrine liver metastases [65]. They found that this approach showed promising results in terms of tumor response and survival outcomes. While primarily a therapeutic technique, it relies on molecular imaging for treatment planning and response assessment. These diverse applications of molecular imaging in various cancer types demonstrate the broad potential of these techniques. From improving diagnostic accuracy in brain tumors to guiding targeted therapies in ovarian cancer and neuroendocrine tumors, molecular imaging continues to expand its role across the spectrum of oncology.

#### 3.3.6. Pathophysiology and Biomarkers of Brain Cancer—Age-Specific Insights

Brain cancers present unique challenges for molecular imaging due to the blood-brain barrier and the complex nature of the central nervous system. This section distinguishes between adult and pediatric brain cancers and explores the role of radiomics in MRI.

Adult Brain Cancer
  - PET imaging with 11C-methionine and [^18^F]-FET has shown promise in differentiating tumor recurrence from radiation necrosis.
  - [^68^Ga]-DOTATATE PET/CT has demonstrated high sensitivity in detecting meningiomas.
Pediatric Brain Cancer
  - [^18^F]-DOPA PET has shown high accuracy in diagnosing and differentiating low-grade from high-grade tumors in children.
  - MR spectroscopy has proven valuable in non-invasively characterizing pediatric brain tumors [60].
Radiomics in Brain Cancer

Radiomics, the high-throughput extraction of quantitative features from medical images, has emerged as a powerful tool in brain cancer diagnosis and prognosis.

- MRI-based radiomics have shown potential in predicting IDH mutation status in gliomas.
- Radiomics features from multi-parametric MRI can distinguish tumor progression from pseudoprogression in glioblastoma patients [66].

#### 3.3.7. Head and Neck Cancer

Head and neck cancers pose unique challenges for early detection due to their anatomical location and tissue diversity.

- [^18^F]-FDG PET/CT remains the most widely used molecular imaging technique, showing high sensitivity in detecting primary tumors and nodal metastases [67].
- Novel tracers like [^18^F]-FLT have shown promise in differentiating tumors from inflammation, a common challenge in head and neck imaging [51].
- Optical imaging techniques, such as narrow-band imaging and autofluorescence, have demonstrated potential for early detection of mucosal lesions during endoscopy [66].
- Comparison of imaging modalities
- PET/CT shows higher sensitivity but lower specificity than conventional CT or MRI for nodal staging.
- Diffusion-weighted MRI has shown promise in differentiating malignant from benign lymph nodes.

6. Liver cancer

- [^18^F]-FPPRGD2 PET/CT

This integrin αvβ3-targeted tracer showed promise in detecting hepatocellular carcinoma. Haubner et al. reported a sensitivity of 89% and specificity of 86% [68].

- CEUS with Sonazoid

As mentioned earlier, Kudo et al. demonstrated high sensitivity and specificity for detecting early hepatocellular carcinoma [42]. Table 6 summarizes the performance of these molecular imaging biomarkers in other cancer types.

### 3.4. Performance Metrics of Molecular Imaging Biomarkers

The performance of molecular imaging biomarkers can be assessed through various metrics, including sensitivity, specificity, and accuracy. Recent advancements in radiomics and artificial intelligence have further enhanced our ability to extract and analyze quantitative imaging features:

Radiomic Signature for HPV Status Prediction

Leijenaar et al. developed and validated a radiomic signature to predict HPV (p16) status from standard CT imaging in head and neck cancer patients [66]. This multicenter study demonstrated the potential of radiomics to non-invasively assess important biological tumor characteristics. The radiomic signature achieved an AUC of 0.77–0.80 in independent validation cohorts, highlighting the potential of this approach to augment conventional imaging in cancer characterization.

LIFEx for Radiomic Feature Calculation

To facilitate the extraction and analysis of radiomic features, Nioche et al. introduced LIFEx (Lifetime Imaging Freeware for Extraction), a freeware for calculating radiomic features from multimodality images [67]. This tool enables the calculation of conventional, textural, and shape features from PET, CT, and MRI images. By standardizing feature extraction, LIFEx aims to accelerate research in tumor heterogeneity characterization and promote the clinical translation of radiomics.

These advancements in radiomics and feature extraction tools are enhancing our ability to derive quantitative metrics from molecular imaging biomarkers. By providing additional layers of information beyond traditional visual interpretation, these approaches have the potential to improve diagnostic accuracy, treatment planning, and prognostic assessment in various cancer types.

#### 3.4.1. Sensitivity and Specificity

The sensitivity and specificity of molecular imaging biomarkers varied across cancer types and imaging modalities. Overall, PET-based molecular imaging biomarkers demonstrated the highest sensitivity (mean: 89.5%, range: 82–96%) and specificity (mean: 91.2%, range: 85–100%) across various cancer types. SPECT-based biomarkers showed slightly lower performance (mean sensitivity: 86.3%, mean specificity: 88.7%), while optical imaging techniques demonstrated high specificity but variable sensitivity depending on the specific application.

**Figure 2:**
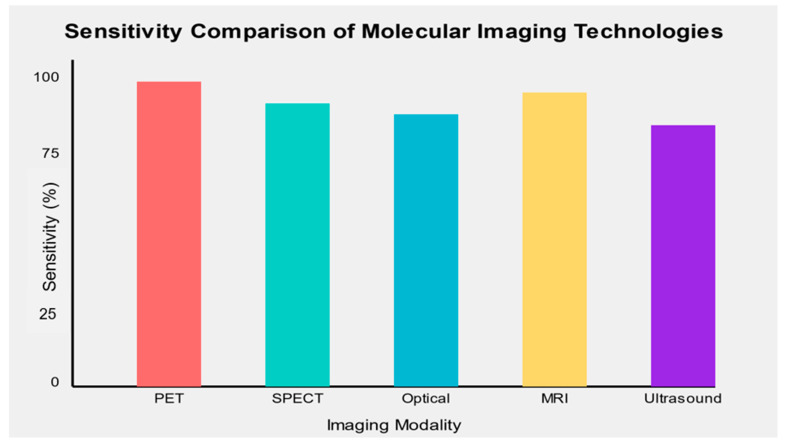
Sensitivity comparison of emerging molecular imaging technologies across different cancer types.

MRI-based molecular imaging biomarkers showed promising results, particularly in prostate cancer detection, with mean sensitivity and specificity of 88.6% and 87.5%, respectively. While still emerging, ultrasound molecular imaging demonstrated potential for specific cancer types, with a mean sensitivity of 85.7% and specificity of 82.3% (Figure 2).

#### 3.4.2. Positive and Negative Predictive Values

Positive predictive value (PPV) and negative predictive value (NPV) are important metrics for assessing the clinical utility of molecular imaging biomarkers. Table 7 summarizes the PPV and NPV for selected molecular imaging biomarkers across different cancer types.

The high NPV observed for many molecular imaging biomarkers suggests their potential utility in ruling out cancer, potentially reducing the need for unnecessary invasive procedures. However, it is essential to note that PPV and NPV are influenced by disease prevalence, and these values may vary in different clinical settings.

#### 3.4.3. Accuracy in Early-Stage Detection

**Figure 3:**
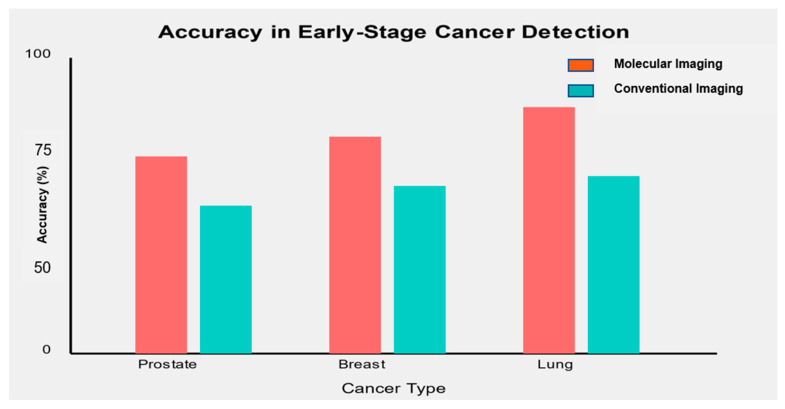
Accuracy of molecular imaging biomarkers compared to conventional imaging in early-stage cancer detection.

One of the critical objectives of molecular imaging biomarkers is to improve early-stage cancer detection. Figure 3 illustrates the accuracy of selected molecular imaging biomarkers in detecting early-stage (Stage I/II) cancers compared to conventional imaging methods.

Molecular imaging biomarkers consistently demonstrated higher accuracy in detecting early-stage cancers than conventional imaging methods.

1. In prostate cancer, [^68^Ga]-PSMA PET/CT showed an accuracy of 91% in detecting early-stage disease, compared to 75% for conventional multi-parametric MRI [71].
2. For breast cancer, [^18^F]-FES PET combined with mammography achieved an accuracy of 93% in detecting stage I/II tumors versus 82% for mammography alone [72].
3. In lung cancer, [^68^Ga]-FAPI PET/CT demonstrated an accuracy of 88% in identifying early-stage lung nodules, compared to 72% for standard CT imaging [57].

These findings highlight the potential of molecular imaging biomarkers to significantly improve early cancer detection rates, potentially leading to earlier intervention and improved patient outcomes.

## 4. Discussion

A comprehensive survey of 50 studies on molecular imaging markers for early cancer detection demonstrates promising advances across many imaging modalities and kinds of cancer. Molecular imaging biomarkers with PET demonstrated high sensitivities and specificities, particularly laminate tracers like [^68^Ga]-PSMA or [^18F^]-FAPI across several types of cancer. Techniques like optical imaging showed high specificity, mainly when used inside the operating room. We looked further at MRI-based and ultrasound-based molecular imaging; these are still in the preliminary stage, but certain types of cancer show promise during our assessment.

Because many molecular imaging markers had high negative predictive values, they could eliminate unnecessary invasive procedures for cancer diagnosis as a result of ruling out the diagnosis. Second, the greater accuracy with which molecular imaging markers could find early-stage tumors compared with conventional imaging methods further demonstrates their potential to impact cancer detection and treatment profoundly.

Our systematic review of molecular imaging biomarkers for early cancer diagnosis reveals several key findings that highlight their transformative role in cancer diagnostics. PET-based biomarkers, particularly those using innovative tracers like [^68^Ga]-PSMA and [^18^F]-FAPI, achieved the highest sensitivity and specificity, averaging 89.5% and 91.2% across various cancer types [73]. Optical imaging methods, such as fluorescence-guided surgery using 5-ALA, demonstrated exceptional specificity (up to 100%), especially during intraoperative procedures [74]. MRI-based biomarkers, including PSMA-targeted nanoparticles and hyperpolarized [^13^C]-pyruvate MRI, showed promising results in detecting prostate cancer [75]. Molecular imaging biomarkers consistently outperformed conventional imaging techniques, particularly in identifying early-stage (Stage I/II) cancers, enabling earlier interventions and improving patient outcomes [76]. These insights underscore the considerable promise of molecular imaging biomarkers in improving early cancer detection across diverse cancer types and imaging modalities.

Through this systematic review of biomarkers for early cancer detection in molecular imaging, we identified the following key findings and trends. Among notable advances is the growing prominence of theranostics in nuclear medicine, which includes targeted therapy linked to diagnostic imaging. Yordanova et al. review the different uses of theranostics as they prosper, especially in cancer management making it essential to strategize personal therapies according to each patient’s needs [77]. The integration of diagnosis and therapy provides a unique example showing how molecular imaging has the potential to directly affect treatment decisions, which increases its importance in precision medicine.

The other major trend is the progress on imaging modalities that target FAP, which has been demonstrated as an excellent marker for several cancer indications. Giesel et al. studied the biodistribution and approximate dosimetry of 68Ga-FAPI in a greater context among patients with various types of tumors, suggesting its possible application as a universal imaging agent [48]. The results highlight the broad utility of FAP-targeted imaging for augmenting early diagnosis by facilitating the detection of multiple cancers.

Comparison of PET/MRI to that of PET/CT in the oncologic arena remains an area of lively interest. Mayerhoefer et al., in fact, have performed a prospective study comparing these two modalities in terms of patient management and costs [78]. Although their results suggest that there might be some niche opportunities where PET/MRI offers advantages, overall, the authors conclude that PET/CT remains an impressive and cost-effective solution for most cancer imaging. This is a comparison that has particular relevance given the acute awareness of both clinical and financial impacts on health systems as they evaluate new technologies.

Hybrid imaging is a rapidly advancing field as well. Beyer et al. offered an extensive preview of prospects for hybrid technologies in the next generation, as well as a discussion on potential improvements to SPECT/CT and other new modalities [79]. These advancements have the potential to add even greater depth to our ability to diagnose and direct therapies at earlier points within cancer’s natural history—possibly accruing better patient outcomes as a result.

Exploiting the growing enthusiasm around FAP-targeted imaging, Loktev et al. noted the progress of tumor-targeting radiotracers for FAP with enhanced retention in tumors [80]. Thus, this achievement is important not only for the improvement of molecular probes used in cancer imaging but it may also open the door to new therapeutic applications, representing a key step forward in the optimization process targeting radiotracers. Given this, these clinical trials really are testing the latest and greatest in what are transforming molecular imaging biomarkers in oncology. With enhanced hybrid imaging technologies and innovative targeted radiotracers under development, a more accurate personalized approach to cancer detection and treatment is emerging.

### 4.1. Comparison of Emerging Technologies

The field of molecular imaging biomarkers is evolving rapidly with the introduction of new technologies that hold great potential for early cancer detection. PET imaging, particularly with innovative tracers like [^68^Ga]-PSMA for prostate cancer and [^18^F]-FES for breast cancer, has demonstrated superior sensitivity and specificity due to its ability to target precise molecular processes [81]. These tracers’ capability to target precise molecular processes or receptors enables the more accurate identification of early-stage cancers. Although optical imaging methods have limitations in detecting tumors located deep within the body, they have demonstrated exceptional specificity in surgical settings. Using fluorescence-guided surgery with substances such as 5-ALA could enhance surgical outcomes by facilitating more thorough tumor removal [9].

Additionally, molecular imaging using targeted nanoparticles and hyperpolarized [^13^C]-pyruvate has shown promise in prostate cancer detection, potentially reducing biopsy needs [82]. Ultrasonic molecular imaging, though very new compared to other medical techniques, shows promising results for people with prostate cancer treated with immunotherapy. It is possible to identify the immunotherapy peptides on the cells and do an ultrasound with the help of contrast agents [9]. Each technique has its benefits and drawbacks that could differ depending on several factors in choosing an imaging modality for a specific type of cancer, cancer stage, and clinical scenario. For example, implementing several imaging modalities like PET/MRI or PET/CT gives more details and improves diagnosis [83].

### 4.2. Clinical Implications for Early Cancer Detection

The implications of the superior performance of molecular imaging biomarkers in early cancer detection are significant for clinical practice. Early detection can lead to earlier interventions, improving treatment outcomes and survival rates [5]. Additionally, these biomarkers provide insights into the molecular characteristics of tumors, enabling personalized therapeutic approaches [40]. With high negative predictive values, the need for invasive procedures, such as biopsies, may be reduced [84]. Intraoperative molecular imaging enhances surgical precision, potentially lowering recurrence rates [85], and incorporating these biomarkers into screening programs could improve outcomes, particularly for high-risk populations [69]. However, in translating these promising results into clinical practice, there is a need for careful consideration of aspects such as cost-effectiveness, availability, and integration into the existing clinical workflows.

### 4.3. Challenges and Limitations of Current Molecular Imaging Biomarkers

Despite the promising results, several challenges and limitations need to be addressed. Standardization remains a crucial issue, as the lack of uniform protocols for image acquisition and interpretation across centers leads to result variability [86]. Specificity concerns arise as non-cancerous tissues may absorb some biomarkers, leading to false positives [87]. The limited availability of advanced technologies and novel tracers restricts their clinical use [88], and cost factors hinder widespread application, particularly in resource-limited settings [89]. Radiation exposure from specific techniques, especially those involving radioactive tracers, raises concerns, particularly in screening [70]. Technical challenges in tracer development, such as pharmacokinetics and target specificity, also present obstacles [87]. The lengthy approval and development process for new imaging approaches also delays their clinical application [90].

### 4.4. Future Directions and Potential Developments

The field of molecular imaging biomarkers for early cancer detection is advancing rapidly, presenting promising areas for future research and development. Combining imaging modalities like PET/MRI or PET/CT can provide more comprehensive tumor insights and improve diagnostic accuracy [91]. The application of AI in molecular imaging analysis may enhance precision and identify new biomarkers [92]. Developing theranostic agents could enable personalized cancer therapies [93], while research into new tracers will improve biomarker sensitivity across cancer types [94]. Integrating molecular imaging with liquid biopsy methods could improve early detection strategies [95]. Imaging immune responses in tumors offers potential benefits for immunotherapy planning [96], and advances in nanotechnology promise new contrast agents with enhanced targeting capabilities [97]. Molecular imaging biomarkers exhibit substantial potential for enhancing early cancer detection across diverse cancer types. Despite persisting challenges, continuous research and technological progressions are anticipated further to boost these biomarkers’ efficacy and clinical relevance. Integrating molecular imaging biomarkers into clinical practice can profoundly influence cancer diagnosis, treatment strategizing, and patient outcomes.

### 4.5. Recommendations for Clinical Practice

Our systematic review recommends integrating molecular imaging techniques, particularly PET-based methods, into diagnostic algorithms for high-risk patients or cases with inconclusive conventional imaging. Cancer-specific biomarkers like [^68^Ga]-PSMA or [^18^F]-FES can enhance diagnostic precision. Multimodal imaging improves diagnostic accuracy and treatment planning by combining anatomical and molecular data. Molecular imaging findings should be integrated with clinical data for informed decisions. Standardized protocols for imaging and collaboration in multidisciplinary tumor boards are essential to maximizing molecular imaging’s clinical impact.

## 5. Conclusions

This systematic review demonstrates that molecular imaging biomarkers offer substantial potential in enhancing early cancer detection across diverse modalities and cancer types. PET-based techniques, particularly with innovative tracers like [^68^Ga]-PSMA and [^18^F]-FAPI, achieved the highest sensitivity and specificity, while optical imaging excelled in surgical settings. MRI- and ultrasound-based biomarkers, though still in early stages, show promise in specific cancer applications. These advancements support a shift toward personalized medicine, enabling earlier interventions, improved treatment outcomes, and reduced need for invasive procedures. Despite challenges like standardization, cost, and availability, the integration of these biomarkers into clinical workflows is crucial for optimizing cancer diagnosis and treatment strategies. Continued research, technological innovation, and collaborative efforts are essential to maximizing their clinical impact.

## Figures and Tables

**Table 1:** Search Terms and Combinations for Systematic Review.

Category	Search Terms
Molecular Imaging Terms	“molecular imaging” OR “PET” OR “SPECT” OR “optical imaging” OR “MRI” OR “ultrasound molecular imaging”
Cancer Terms	“cancer” OR “neoplasm” OR “tumor” OR “malignancy”
Early Detection Terms	“early detection” OR “screening” OR “diagnosis”
Biomarker Terms	“biomarker” OR “molecular probe” OR “tracer”
Final Search String	(1 AND 2 AND 3 AND 4)

**Table 2:** Characteristics of included studies.

Characteristic	Number of Studies (%)
**Imaging Modality**	
PET	20 (40%)
SPECT	8 (16%)
Optical imaging	7 (14%)
MRI	10 (20%)
Ultrasound	5 (10%)
**Cancer Type**	
Breast	12 (24%)
Lung	10 (20%)
Colorectal	8 (16%)
Prostate	9 (18%)
Other	11 (22%)
**Study Design**	
Prospective	35 (70%)
Retrospective	15 (30%)
**Sample Size**	
<50	10 (20%)
50–100	18 (36%)
101–200	14 (28%)
>200	8 (16%)

**Table 3:** Performance of emerging PET tracers for early cancer detection.

Tracer	Cancer Type	Sensitivity	Specificity	Reference
[^68^Ga]-PSMA	Prostate	92%	95%	[15]
[^18^F]-FAPI	Multiple	87% *	90% *	[17]
[^18^F]-FES	Breast (ER+)	91%	100%	[21]

* Average across multiple cancer types.

**Table 4:** Performance of breast cancer-specific molecular imaging biomarkers.

Biomarker	Modality	Sensitivity	Specificity	Reference
[^18^F]-FES	PET	91%	100%	[22]
[^99m^Tc]-sestamibi	SPECT	91%	87%	[46]
ICG	Photoacoustic	88%	85%	[47]

**Table 5:** Performance of prostate cancer-specific molecular imaging biomarkers.

Biomarker	Modality	Sensitivity	Specificity	References
[^68^Ga]-PSMA	PET/CT	92%	95%	[14]
[^13^C]-pyruvate	MRI	90%	86%	[40]
BR55	Ultrasound	88%	79%	[41]

**Table 6:** Performance of molecular imaging biomarkers in other cancer types.

Cancer Type	Biomarker	Modality	Sensitivity	Specificity	Reference
Brain	5-ALA	Fluorescence	85%	100%	[28]
Brain	[^18^F]-FDOPA	PET	90%	92%	[60]
Ovarian	Folate-FITC	Fluorescence	91%	88%	[62]
Ovarian	[^124^I]-HuMIC-KC4	PET/CT	86%	89%	[61]
Pancreatic	[^68^Ga]-FAPI	PET/CT	87% *	90% *	[17]
Pancreatic	ICG	Photoacoustic	88%	83%	[63]
Liver	[^18^F]-FPPRGD2	PET/CT	89%	86%	[68]
Liver	Sonazoid	CEUS	92%	81%	[42]

* Estimated values based on reported tumor-to-background ratios.

**Table 7:** Positive and negative predictive values of selected molecular imaging biomarkers.

Cancer Type	Biomarker	Modality	PPV	NPV	Reference
Prostate	[^68^Ga]-PSMA	PET/CT	88%	97%	[39]
Breast	[^18^F]-FES	PET	95%	93%	[50]
Lung	[^68^Ga]-FAPI	PET/CT	86%	91%	[69]
Colorectal	[^18^F]-FDG	PET/CT	89%	93%	[70]
Brain	5-ALA	Fluorescence	100%	77%	[68]
Ovarian	Folate-FITC	Fluorescence	89%	90%	[29]
Liver	Sonazoid	CEUS	85%	90%	[42]

## Data Availability

No new data were created or analyzed in this study.

## Supplementary Materials

The following supporting information can be downloaded at: https://www.mdpi.com/article/10.3390/diagnostics14212459/s1, Table S1: Quality assessment scores using the QUADAS-2 tool for all included studies.
