# Supplementary material for: Molecular Imaging Biomarkers for Early Cancer Detection: A Systematic Review of Emerging Technologies and Clinical Applications
Source: Diagnostics (Basel). 2024 Nov 3;14(21):2459. doi: 10.3390/diagnostics14212459 (PMC11545511; doi:10.3390/diagnostics14212459)
Supplement: Supplementary file 1 [file diagnostics-14-02459-s001.zip › diagnostics-3226060-supplementary.pdf]

Table S1: Quality assessment scores using the QUADAS-2 tool for all included studies

Author (Year)	Risk of Bias				Applicability Concerns			Overall Quality
	Patient Selection	Index Test	Reference Standard	Flow and Timing	Patient Selection	Index Test	Reference Standard	
Chae, S. Y. et al. (2019)	Low	Low	Low	Low	Low	Low	Low	High
Kirienko, M. et al. (2018)	Low	Low	Low	Unclear	Low	Low	Low	Moderate
Joshi, B. P., & Wang, T. D. (2018)	Low	Low	Low	Low	Low	Low	Low	High
Grubmüller, B. et al. (2018)	Low	Low	Low	Low	Low	Low	Low	High
Willmann, J. K. et al. (2017)	Low	Unclear	Low	Low	Low	Low	Low	Moderate
Kratochwil, C. et al. (2019)	Low	Low	Low	Low	Low	Low	Low	High
Kratochwil, C. et al. (2019)	Low	Low	Low	Low	Low	Low	Low	High
Stummer, W. et al. (2006)	Low	Low	Low	Low	Low	Low	Low	High
Piccardo, A. et al. (2016)	High	Low	Low	Unclear	Low	Low	Low	Moderate
Jones, K. M. et al. (2018)	Low	Unclear	Low	Low	Low	Low	Low	Moderate
Hoffmann, M. A. et al. (2018)	Low	Low	Low	Low	Low	Low	Low	High
Sheikhbahaei, S. et al. (2017)	High	Low	Low	Unclear	Low	Low	Low	Moderate
Kiesslich, R. et al. (2007)	Low	Low	Low	Low	Low	Low	Low	High
Sharma, S. K. et al. (2016)	Low	Low	Unclear	Low	Low	Low	Unclear	Moderate
Valluru, K. S. et al. (2016)	Low	Unclear	Low	Low	Low	Low	Low	Moderate
Catalano, O. A. et al. (2017)	Low	Low	Low	Low	Low	Low	Low	High
Hofman, M. S. et al. (2020)	Low	Low	Low	Low	Low	Low	Low	High
Piccardo, A. et al. (2019)	High	Low	Low	Unclear	Low	Low	Low	Moderate
Fendler, W. P. et al. (2017)	Low	Low	Low	Low	Low	Low	Low	High
Kogan, F. et al. (2013)	Low	Low	Low	Low	Low	Low	Low	High
Patel CB. et al. (2018)	Low	Low	Low	Low	Low	Low	Low	High
Yao, Y. et al. (2020)	Low	Unclear	Low	Low	Low	Low	Low	Moderate

Author (Year)	Risk of Bias				Applicability Concerns			Overall Quality
Giovanella, L. et al. (2020)	High	Low	Low	Unclear	Low	Low	Low	Moderate
Conners, A. L. et al. (2017)	Low	Low	Low	Low	Low	Low	Low	High
Kasivisvanathan, V. et al. (2018)	Low	Low	Low	Low	Low	Low	Low	High
Wu, L. et al. (2020)	Low	Low	Unclear	Low	Low	Low	Unclear	Moderate
Sollini, M. et al. (2019)	Low	Low	Low	Low	Low	Low	Low	High
Wang D. et al. (2022)	High	Low	Low	Unclear	Low	Low	Low	Moderate
Chen, H. et al. (2020)	Low	Low	Low	Low	Low	Low	Low	High
Zhao, Y. et al. (2018)	Low	Unclear	Low	Low	Low	Low	Low	Moderate
Afshar-Oromieh, A. et al. (2017)	Low	Low	Low	Low	Low	Low	Low	High
Siva, S. et al. (2019)	High	Low	Low	Unclear	Low	Low	Low	Moderate
Pillay V. et al. (2022)	Low	Low	Low	Low	Low	Low	Low	High
Verger, A. et al. (2018)	High	Low	Low	Unclear	Low	Low	Low	Moderate
Jiang SQ. et al. (2020)	High	Low	Low	Unclear	Low	Low	Low	Moderate
O’Brien. et al. (2024)	Low	Low	Low	Low	Low	Low	Low	High
Gallagher, F. A. et al. (2018)	Low	Unclear	Low	Low	Low	Low	Low	Moderate
Jain, M. et al. (2019)	Low	Low	Low	Low	Low	Low	Low	High
Yordanova et al. (2017)	Low	Low	Low	Low	Low	Low	Low	High
Cui, Y. et al. (2018)	Low	Low	Low	Low	Low	Low	Low	High
Giesel, F. L. et al. (2019)	Low	Low	Low	Low	Low	Low	Low	High
Mayerhoefer, M. E. et al. (2020)	High	Low	Low	Unclear	Low	Low	Low	Moderate
Nioche, C. et al. (2018)	Low	Low	Low	Low	Low	Low	Low	High
Beyer T.. et al. (2011)	Low	Unclear	Low	Low	Low	Low	Low	Moderate
Giesel, F. L. et al. (2018)	Low	Low	Low	Low	Low	Low	Low	High
Leijenaar, R. T. et al. (2018)	High	Low	Low	Unclear	Low	Low	Low	Moderate
Piccardo, A. et al. (2020)	Low	Low	Low	Low	Low	Low	Low	High
Frilling, A. et al. (2020)	Low	Low	Low	Low	Low	Low	Low	High
Loktev, A. et al. (2019)	Low	Low	Low	Low	Low	Low	Low	High

**Author (Year)**	**Risk of Bias**				**Applicability Concerns**			**Overall Quality**
Zeng, Y. et al. (2019)	Low	Low	Low	Low	Low	Low	Low	High
